# How the Prohormone Theory Solved Two Important Controversies in Hormonal and Neural Peptide Biosynthesis

**DOI:** 10.3389/fendo.2013.00148

**Published:** 2013-10-23

**Authors:** Michel Chrétien

**Affiliations:** ^1^Functional Endoproteolysis Laboratory, Institut de Recherches Cliniques de Montréal, Ottawa Hospital Research Institute, Montreal, QC, Canada

**Keywords:** prohormone theory, proprotein convertases, peptide hormones, neuropeptides, biosynthesis

## Abstract

This Prohormone Theory was simultaneously proposed in 1967 by two independent groups using two different approaches and two experimental models. Donald Steiner, in elegant pulse-chase experiments, proposed the existence of proinsulin when he observed that a human insulinoma was producing higher MW forms of immunoreactive insulin, subsequently transformed into insulin-like material (1). Simultaneously and independently, Michel Chrétien, based on amino acid sequence homologies between three pituitary peptides, β-lipotropic hormone (β-LPH), γ-LPH, and β-melanocyte-stimulating hormone (β-MSH), concluded that active peptide hormones are derived from endoproteolytic cleavages of inactive precursors, apparently at pairs of basic amino acids (2). One year later, Donald Chance confirmed that the cleavage sites in proinsulin were also made of paired basic amino acids (3). This novel paradigm solved two major controversies on the biosynthesis of both insulin and neuropeptides. This short review describes how.

## The Insulin Saga

In the mid 1950s and early 1960s, many scientists wandered how insulin was synthesized in the pancreatic β-cells. Fred Sanger had established that insulin is made of two peptide chains linked by disulfide bridges (4). A prevailing view was that insulin was biosynthesized as two separate peptide chains “zipped” post-transcriptionally by interchain disulfide bridges. Preceding that period, Oliver Smithies, as mentioned in his 2007 Nobel Lecture, was looking for a precursor to insulin “which I never found” (5). Many groups in the US, Canada, China, and Germany, using separate insulin A and B chains, had tried to reconstitute insulin *in vitro*, with minimal yield (6). In the mid-1960s, some *in vitro* studies of pancreatic islet tissue had led to the conclusion that the two insulin chains were biosynthesized as separate entities. The controversy was definitely solved, when the amino acid sequence of proinsulin unequivocally proved that insulin is made as a single polypeptide, subsequently modified to its active form by endoproteolysis at pairs of basic residues (3).

## The Neuropeptide Saga

The β-LPH/γ-LPH/β-MSH model of biosynthesis, initially proposed in 1967 for pituitary peptides, contained elements that would solved the upcoming controversy on neuropeptide biosynthesis. In 1969, the field of neuroendocrinology underwent a revolution when Roger Guillemin published the astonishing discovery that thyrotropin releasing factor (TRF) was a tripeptide (7). It was suggested that this tripeptide, like glutathione, was produced by a soluble non-ribosomal enzymatic mechanism and the existence of a TRH synthetase was seriously considered.

The first indication that β-LPH could be a neuropeptide precursor came when Hughes and Kosterlitz published the amino acid sequence of met-enkephalin and noted that it corresponded to residues 61–65 of β-LPH (8). Shortly thereafter, many groups revealed that the main opioid secretory product was the fragment 61–91 of β-LPH, now universally known as β-endorphin, a strong indication that β-LPH (1–91) was its most plausible precursor candidate. Definite proof that β-LPH is the precursor of β-endorphin came about when it was unequivocally demonstrated that β-endorphin is produced by endoproteolytic cleavage of β-LPH at pair basic residues 59–60 (9).

**Figure 1:**
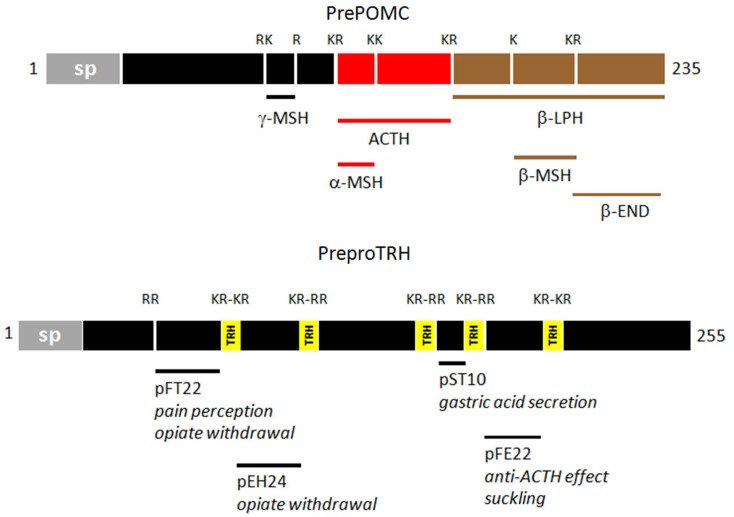
**Diagrammatic representations of prePOMC and preproTRH**. The diagrams are based on rat sequences. The additional functional peptides of proTRH have been reviewed by Nillni and Sevarino (21). Single and paired basic residues (K/R) flanking the functional peptides are shown. (sp: signal peptide).

Coincidently, it was realized that β-LPH itself is part of a larger precursor containing ACTH. The existence of this precursor was confirmed with the cloning of its cDNA; in it, the sequences of its active end products (β-endorphin, MSHs, and ACTH) are flanked by the canonical pairs of basic residues (10). The precursor, now named proopiomelanocortin (POMC) (11), has become the gold standard of endocrine and neuroendocrine precursors. Soon after, the cloning of cDNAs for the other neuropeptides confirmed that all of them were produced through a similar mechanism (12). The non-ribosomal enzymatic concept of TRH production thus became obsolete. The endoproteolysis of polyproteins like ProTRF and POMC (Figure 1) greatly amplifies the multiple active end products of large precursor molecules (13).

During the following decades, post-translational endoproteolytic activation became applicable to numerous other polyproteins, including precursors to neurotrophins, growth factors, transcription factors, receptors, extracellular matrix proteins, bacterial toxins, viral glycoproteins, etc. It is now recognized as a fundamental cellular process, affecting many biological functions and opened a new chapter in biology (13, 14).

The 1967 Prohormone Theory and its biological ramifications implied the existence of endoproteolytic enzymes dedicated to the process (15). These were discovered 23 years later (16, 17). Collectively, they are called proprotein convertases (PCs) or proproteins convertases subtilisin/kexin type (PCSKs). They are calcium-dependent serine endoproteases, structurally related to bacterial Subtilisin and to yeast Kexin (13, 18, 19). The first two, PC1/3-PC2 are considered the prototypical convertases for prohormones and proneuropeptides.

## Conclusion

In solving two major controversies concerning the biosynthetic pathways of insulin and neuropeptides, the 1967 prohormone theory has become a tenet of the peptidergic systems in endocrinology and neuroendocrinology. This is one of many other examples in biology whereby incompatible hypotheses are clarified by one type of results. One of the most famous is certainly the 1943 fluctuation test of Salvador Luria and Max Delbruck (20). Although less spectacular than the genetics of bacterial resistance, the prohormone concept ended two scientific debates and led to new horizons which surpassed all the most elaborate expectations.

## Conflict of Interest Statement

The author declares that the research was conducted in the absence of any commercial or financial relationships that could be construed as a potential conflict of interest.
